# Intraclass correlation values for adolescent health outcomes in secondary schools in 21 European countries

**DOI:** 10.1016/j.ssmph.2016.03.005

**Published:** 2016-04-18

**Authors:** N. Shackleton, D. Hale, C. Bonell, R.M Viner

**Affiliations:** a Centre of Methods and Policy Application in the Social Sciences (COMPASS), University of Auckland, Auckland, New Zealand; b General and Adolescent Paediatrics, Population, Policy & Practice Programme, Institute of Child Health, University College London, United Kingdom; c Department of Social Science, Institute of Education, University College London, United Kingdom

**Keywords:** Intra-class correlation, Schools, Adolescents, Substance use, Mental health

## Abstract

**Background:**

Cluster randomised controlled trials (CRCTs) are increasingly used to evaluate the effectiveness of interventions for improving health. A key feature of CRCTs is that individuals in clusters are often more alike than individuals in different clusters, irrespective of treatment. This similarity within clusters needs to be taken into account when planning CRCTs to obtain adequate sample sizes, and when analysing clustered data to obtain correct estimates.

**Methods:**

Nationally representative data from 15 to 16 year olds were analysed, from 21 of the 35 countries that participated in the 2007 European School Survey Project on Alcohol and Other Drugs. Within country school level intra-class correlation coefficients (ICCs) were calculated for substance use (self-reported alcohol use, regular alcohol use, binge drinking, any smoking, regular smoking, and illicit drug use) and psychosocial health (depressive mood and self-esteem). Unadjusted and adjusted ICCs are presented. ICCs are adjusted for student sex and socioeconomic status.

**Results:**

ICCs ranged from 0.01 to 0.21, with the highest (0.21) reported for regular smoking. Within country school level ICCs varied substantially across health outcomes, and among countries for the same health outcomes. Estimated ICCs were consistently higher for substance use (range 0.01–0.21), than for psychosocial health (range 0.01–0.07). Within country ICCs for health outcomes varied by changes in the measurement of particular health outcomes, for example the ICCs for regular smoking (range 0.06–0.21) were higher than those for having smoked at all in the last month (range 0.03–0.17).

**Conclusions:**

For school level ICCs to be effectively utilised in informing sample size requirements for CRCTs and adjusting estimates from meta-analyses, the school level ICCs need to be both country and outcome specific.

## Highlights

• School level ICCs in substance use and psychosocial health are presented for 21 European countries.
• Larger ICCs are estimated for substance use than for psychosocial health.
• There is large variation in ICC׳s within countries between outcomes, and between countries within outcomes.

## 1 Background

Cluster randomised controlled trials (CRCTs) are increasingly used to evaluate the effectiveness of interventions for improving health (Bland, 2004, Klar and Donner, 2001). CRCTs involve the random assignment of whole clusters, such as schools, hospitals, clinics or communities, rather than individuals (Raudenbush, 1997). CRCTs are particularly useful where researchers are specifically interested in the cluster, as it may not be feasible to randomly assign individuals to clusters such as schools or hospitals, or where they are interested in the cluster-level effects of an intervention. The advantages and disadvantages of using CRCTs have been discussed in detail in a series of publications by Donner and Klar (2001/2002/2004) (Donner and Klar, 2002, Donner and Klar, 2004, Klar and Donner, 2001). A key feature of CRCTs is that individuals in clusters are often more alike than individuals in different clusters, irrespective of treatment. This similarity within clusters needs to be taken into account when planning CRCTs to obtain adequate sample sizes, and when analysing clustered data to obtain correct estimates. The focus of this paper is on presenting estimates of the similarity of health outcomes of students within schools across a large number of European countries.

Students in the same school are more similar, on average, than students selected from different schools. This is true for a range of educational and health outcomes (McKenzie, Ryan, & Di Tanna, 2014). This dependence of individuals within clusters leads to two potential problems. First, CRCTs require more subjects than RCTs to obtain adequate statistical power because observations are not independent. Secondly, the clustering of the data needs to be addressed through the use of appropriate analysis techniques (such as multilevel models), otherwise standard error estimates will be deflated resulting in an increased risk of Type I errors (false positives) (Klar and Donner, 2001, McKenzie et al., 2014).

The intra-class correlation coefficient (ICC) measures the degree of within cluster dependence for a variable, and can therefore be used in power calculations to compute the necessary sample sizes for specific outcomes for CRCTs. If all observations are independent of one another, the ICC will be 0. If all the responses from observations in all clusters are exactly the same, the ICC will be 1. For trials, the greater the value of the ICC, the greater the sample size required (Klar and Donner, 2001, McKenzie et al., 2014, Raudenbush, 1997). To achieve the equivalent power of an individual level randomised un-clustered sample, the sample size has to be inflated by the design effect:

Design Effect = 1+(*m*−1)^*^ICC, where *m* represents the average cluster size.

The ICC can also be used to correct the estimates of analyses that have not taken the clustered nature of the data into account, by either retrospectively inflating the standard errors to account for the dependence, or reducing the sample size (Hedges, 2007, Hedges and Hedberg, 2007). This is potentially very important for research that compares or combines the results of analyses, such as meta-analyses. Hence it is useful to know ICCs in advance of designing CRCTs, to ensure adequate sample size for power, and for adjusting the analysis of clustered data in meta-analysis, where clustering has not been taken into account. Knowledge of ICC׳s is important for a further reason that is often overlooked. When interpreting the impact of school level variables in multilevel models, the lower the value of the ICC, that is the lower the proportion of the variance that is at the school level and therefore the less relevant the school context is, the more likely you are to obtain a significant association between a school-level variable and the outcome (Lagerlund et al., 2015, Merlo, 2015). Researchers need knowledge of ICC׳s to accurately interpret school level variables in multilevel models.

The importance of the ICC has been widely acknowledged for educational outcomes. ICCs are deemed important because they highlight the differential performance of schools (variation between schools) in terms of student achievement, conditional upon prior student achievement (Goldstein, Huiqi, Rath, & Hill., 2000). Estimates of ICCs for educational achievement in the UK range between 0.10 and 0.25, which suggests that between 10% and 25% of the total variance is at the school level (Hedges and Hedberg, 2007, Hale et al., 2014). Where researchers have reported estimates of the ICCs for health related outcomes, the estimated ICCs are significant but smaller in magnitude than for educational outcomes (Bonell et al., 2013, Hale et al., 2014, Sellström and Bremberg, 2006). Hale et al. (2014) reported the ICCs for a range of health outcomes from three large English datasets, with the majority of the ICCs for health outcomes being lower than 0.10, compared to the ICCs for academic achievement which were between 0.19 and 0.25 in the same samples (Hale et al., 2014). Bonell et al. (2013a) performed a systematic review of multilevel school studies from the USA, Canada, the UK, Australia, Thailand, Israel and several European countries. They reported ICCs between 0.02 and 0.14 for smoking and alcohol use, and ICC׳s less than 0.06 for students’ problem behaviour and well-being (Bonell et al., 2013a).

The similarity of students within schools may be due to selection, whereby individuals affiliate with others who have similar attributes to themselves (Simons-Morton & Farhat, 2010). Schools likely attract students with similar characteristics, hence selection into schools results in students having more similar characteristics or behavioural patterns than one would expect if selection into schools was random (Simons-Morton & Farhat, 2010). Alternatively, it may be due to socialisation processes whereby adolescent׳s behavioural patterns become more similar in response to interactions with other students in the same school, and the formation of perceived or actual social norms about behaviours (Simons-Morton and Farhat, 2010).

The terms “compositional effects” and “contextual effects” have also been used to explain the influence of places on individuals’ outcomes (Macintyre, Ellaway, & Cummins, 2002). Compositional effects refer to the influence of the collective properties of the student body on individual student׳s behaviour. For example, some schools will have a predominance of students from socio-economically advantaged families, who are highly motivated and have high levels of prior achievement. This compositional aspect of the school can have a positive influence on achievement for all students in the school (Lauder, 2007, van Ewijk and Sleegers, 2010). Contextual effects refers to the influence of the school itself (such as the physical environment, policies and regulations) on student׳s behaviour (Macintyre et al., 2002). Compositional effects link to the selection and socialisation processes outlined in the previous paragraph. Differential compositions of schools are a product of selection effects into schools (Harker & Tymms, 2004).The influence of school composition on individual student׳s behaviour is partially explained by socialisation processes (Harker & Tymms, 2004).

Markham and Aveyard׳s (2003) theory of human functioning attempts to explain the relationship between schools and student׳s behaviours, placing the emphasis on the contextual explanation (the effect that schools have on students). This theory is rooted in Bernstein׳s (1975) theory of cultural transmission. Schools impart two types of knowledge, the instructional order (acquisition of knowledge and skills), and the regulatory order (appropriate ways of behaving). Students who reject, or are unable to meet the demands of, these kinds of learning subsequently reject the values of the school and affiliate with youth subcultures that are more likely to promote substance use.

Alternatively, the notion of peer contagion effects (Cohen and Prinstein, 2006, Dishion and Tipsord, 2011) and social mimicry (Moffitt, 1993) place emphasis on the compositional elements of the school environment using socialisation processes to explain similarity in behaviours. Peer contagion effects suggests that students influence each other׳s behaviours and emotions, such that deviant behaviours and emotional problems are transmitted from one student to another. The transmission of behaviours is an unintended consequence of social relationships (Cohen and Prinstein, 2006, Dishion and Tipsord, 2011). A related but distinct theory is that of social mimicry, which argues that behaviours are explained through the desire for social acceptance and esteem (Moffitt, 1993).

A number of school factors have repeatedly been shown to protect against unhealthy behaviour and poor mental health, particularly school connectedness or more broadly aspects of the school ‘culture’ and ethos (Bonell et al., 2013, Bonell et al., 2013, Viner et al., 2012). Several systematic reviews of school based interventions show the potential for schools to influence a wide range of student health and behavioural outcomes, including nutrition and activity, substance use, sexual health behaviours, and violence related outcomes (Bonell et al., 2013, Fletcher et al., 2008, Foxcroft and Tsertsvadze, 2011, Langford et al., 2014, Sellström and Bremberg, 2006). School based interventions that address the school environment are effective at changing student health behaviours (Fletcher et al., 2008, Foxcroft and Tsertsvadze, 2011, Langford et al., 2014). Higher ICCs for specific behaviours could suggest that school-level interventions are more effective in changing those behaviours, as a higher proportion of variance at the school-level suggests that the outcome is predicted by characteristics of the school as well as characteristics of the student. Although, this is only true if the ICC is not a reflection of selection effects into schools (Macintyre et al., 2002).

A serious limitation of the current literature on the effectiveness of school level interventions is a reliance on evidence from the US (Bonell et al., 2013, Fletcher et al., 2008, Foxcroft and Tsertsvadze, 2011). There is a clear need for interventions from other countries to contribute to the evidence on school-based interventions. In this paper we use data from the 2007 European School Survey Project on Alcohol and Other Drugs (ESPAD) (Hibell et al., 2009) to provide plausible country-specific estimates of ICCs for a range of adolescent health outcomes in 21 European countries. We test the proportion of variance at the school level in several key health outcomes, including substance use (licit and illicit) and psychosocial wellbeing (depressive mood, self-esteem), where the data are available. We also compare the estimates across countries to determine the extent of differences among countries.

## 2 Methods

### 2.1 Sample

We used data from the 2007 European School Survey Project on Alcohol and Other Drugs (ESPAD) (Hibell et al., 2009). Thirty-five European countries took part in the project. Standard methodological guidelines were implemented to collect data on school students. The target population consisted of 15–16 year old students born in 1991. A sample of at least 2800 students per country was recommended. Details of sampling and survey methods in each country, and other information including response rates and sample representativeness, can be found in Hibell et al. (2009).

We restricted our analyses to 21 countries in which recruited samples were deemed nationally representative (without weighting) by ESPAD, and which included schools in the sampling frame. These were: Armenia, Austria, Britain, Bulgaria, Croatia, Cyprus, Czech Republic, Denmark, Estonia, Finland, Greece, Iceland, Ireland, Lithuania, Malta, Poland, Portugal, Slovakia, Slovenia, Sweden and Switzerland. The proportion of the 1991 cohort still enroled in schools at the age of 15/16 was over 90% in all countries except Bulgaria (78%). Sample sizes ranged from 877 (Denmark) to 6340 students (Cyprus). Twelve countries sampled multiple classes per school, but we did not include a class level in the models due to low numbers of classes per school on average. As shown in Table 1, the number of classes per school ranged from 1.08 to 7.54, with most countries having between one and two participating classes per school. The small number of clusters at the class level coupled with the small sample size per class suggests that estimates of variance at this level would be poor and downwardly biased (Austin, 2010). Also, students of this age group typically do not spend time in a single class formation, but change classes for each subject.

**Table 1:** Observations per unit.

Country	Schools	Students	Classes	Student/schools	Class/school	Student/class
Armenia	270	4055		15.02		
Austria	123	2571	269	20.9	2.19	9.56
Britain	99	2179		22.01		
Bulgaria	210	2353	226	11.2	1.08	10.41
Croatia	118	3008	275	25.49	2.33	10.94
Cyprus	53	6340	421	119.62	7.94	15.06
Czech rep	350	3901		11.15		
Denmark	36	877	69	24.36	1.92	12.71
Estonia	102	2372		23.25		
Finland	299	4988		16.68		
Greece	253	3060	389	12.09	1.54	7.87
Iceland	122	3510	209	28.77	1.71	16.79
Ireland	94	2221		23.63		
Lithuania	132	2411	302	18.27	2.29	7.98
Malta	61	3668		60.13		
Poland	209	2120		10.14		
Portugal	531	3141		5.92		
Slovakia	119	2468	247	20.74	2.08	9.99
Slovenia	119	3085	168	25.92	1.41	18.36
Sweden	175	3179		18.17		
Switzerland	327	2499	383	7.64	1.17	6.52

### 2.2 Measures

#### 2.2.1 Substance use

Self-reported substance use from the last 30 days is used. This is frequently used in international adolescent health and behaviour surveys such as those undertaken by the World Health Organisation (Warren et al., 2000). Self-reported substance use from longer time periods can be subject to greater recall issues. More frequent substance use is more predictive of later harms (Fergusson, Boden, & Horwood, 2006).

##### 2.2.1.1 Alcohol

Students were asked “On how many occasions (if any) have you had any alcoholic beverage to drink during the last 30 days?” Two binary variables were created from this question: any alcohol use in the last 30 days, and regular drinking over the last 30 days (where students report drinking alcohol on at least 6 occasions in the last 30 days).

Binge drinking was defined as reporting one or more occasions of consuming five or more drinks on a single occasion in the last 30 days. The Data from four countries were deemed not comparable with other countries due to alterations of the response categories from categorical to numerical (Austria, Germany), differences in question wording (Ireland), and changes in the volume of alcohol listed as constituting a single drink (Portugal).

##### 2.2.1.2 Smoking

Students self-reported their frequency of smoking over the last 30 days. Two binary outcomes were created that captured whether students smoked at all in the last 30 days and whether students smoked at least once per day for the last 30 days.

##### 2.2.1.3 Illicit drug use

Three measures assessed illicit drug use over the last 30 days: one each regarding cannabis, ecstasy and inhalants. A binary variable was created with responses of 1 or more on any of these items used to indicate any illicit drug use in the last 30 days.

#### 2.2.2 Psychosocial health

Psychosocial health outcomes were taken from an optional module of the ESPAD survey: 9 countries asked students about self-esteem and 11 countries asked students questions relating to depressive mood.

Self-esteem was measured using the ten item Rosenberg׳s Self-esteem scale (Rosenberg, 1965). Items were recoded so that higher scores indicated higher self-esteem. Typical items from the scale include “On the whole, I am satisfied with myself” and “I certainly feel useless at times” with a four point Likert scale for responses (0 strongly agree/1 agree/2 disagree/3 strongly disagree). The scale ranges from 0 to 30. Scores between 15 and 25 are within normal range; scores below 15 suggest low self-esteem. Multiple studies have considered the validity and reliability of this scale finding evidence for good construct validity (Bagley and Mallick, 2001, Greenberger et al., 2003), convergent validity (Hagborg, 1993), and reliability (Greenberger et al., 2003, McCarthy and Hoge, 1982). Typical Cronbach׳s alpha values for this scale are greater than 0.8, suggesting high amounts of internal consistency. The scale also correlates well with measures of mental health (Bagley and Mallick, 2001, Griffiths et al., 1999), and with other measures of self-esteem and self-concept (Hagborg, 1993).

Depressive symptoms were measured using the depressive mood scale, a short form (6 item) of the Centre of Epidemiological Studies Depression-Scale (CES-D) (Radloff, 1977). Students were asked “During the LAST 7 DAYS, how often …(a) have you lost your appetite, you did not want to eat/(b) have you had difficulty in concentrating on what you want to do/(c) have you felt depressed/(d) have you felt that you had to put great effort and pressure to do the things you had to do/(e) have you felt sad/(f) could not you do your work (at home, at work, at school)” with four response options for each item (0 rarely or never/1 sometimes/2 several times/3 most of the time). The scale was coded so that higher scores indicated a more depressive mood.

The full CES-D is a valid and reliable instrument for assessing depressive symptoms (LaChapelle and Alfano, 2005, Miller et al., 2008, Weissman et al., 1977). Scores of 16 or more in the full CES-D indicate clinically significant depression (Weissman et al., 1977). The validity of this short depression scale was evaluated by the ESPAD Research team (Hibell et al., 2009). The short and full CES-D scales were compared in a survey of 5249 adolescents. Cronbach׳s alphas on the short form scale ranged from 0.746 among boys in Flanders (Belgium) to 0.855 among boys in Cyprus. Differences in the relationship between the CES-D scores for the long and short form, and their relationship to the following variables were small and judged to be a satisfactory trade-off for the reduction in burden on the respondents: general satisfaction with life, consulting a doctor for psychological problems in that last 12 months, taking antidepressants under prescription, and attempted suicide (Hibell et al., 2009).

### 2.3 Analyses

Cleaned data were obtained from the ESPAD data bank. Full information on this data cleaning process and on the country specific sampling methodologies is available in the 2007 ESPAD report (Hibell et al., 2009).

First, we assessed the appropriateness of sample sizes by observing the number of students, classes and schools that were observed in each country. We report the country level means of the outcome variables to provide context for interpretation. Likelihood ratio tests were used to assess whether there was a significant amount of within-country between-school level variance. Likelihood ratio test compared two models, a three level model (Country/School/Student) with the school level included, and a two level model without school level included (Country/Student). Intra-class correlation coefficients were also calculated for the three level model to assess the magnitude of the average within country between school variance. These were estimated with 95% confidence intervals to aid comparisons between outcomes.

The intra-class correlation coefficients for this three level model were calculated in the following way:

Similarity of students within the same country:

ICC=var(*ν*0)/[var(*v*0)+var(*u*0)+var(*e*0)]

Similarity of students within the same school in the same country:

ICC= var(*ν*0)+var(*u*0)/[var(*v*0)+var(*u*0)+var(*e*0)],

where var(*ν*0) is the level 3(country) residual variance, var(*u*0) is the level 2 (school) residual variance, and var(e0) is the variance of the level 1 (student) residuals. For the three level models the variance in binary outcomes were estimated using linear probability models.

Where there was a significant amount of within country between school level variance, two level multilevel models (School/students) were run within each country separately to calculate country specific school ICCs. That is, we treated each country as if it were a separate data set.

School ICCs for continuous outcomes were calculated using the following formula:

ICC=var(*u*0)/[var(*u*0)+var(*e*0)],

where var(*u*0) is the level 2 (school) residual variance, and var(*e*0) is the variance of the level 1 (student) residuals.

There are different methods available for calculating ICCs for binary variables (Li et al., 2008, Wu et al., 2012). We chose a method that ensured the ICC estimates are not smaller than 0, and that within cluster variance does not depend on cluster prevalence (Wu et al., 2012). For binary outcomes ICCS were calculated in the following way:

ICC=var(*u*0)/[var(*u*0)+*π*^2^/3],

where var(*u*0) is the level 2 (school) residual variance, and *π*^2^/3 (which is equal to 3.29) is by assumption the variance of the level 1 (student) residuals.

We considered both unadjusted ICCs and ICCs adjusted for characteristics of the students. The unadjusted ICCs are useful for the power calculations used in planning CRCTs. The models were then adjusted for student׳s sex and socioeconomic status which was measured via students reports of mothers’ and fathers’ education (Completed primary school or less/Some secondary school/Completed secondary school/ Some college or university/Completed college or university). As students were all of similar ages (15–16) we did not adjust for age. Ethnicity (or suitable proxy׳s) was not available in the dataset.

## 3 Results

Table 1 breaks down the number of observations for every possible clustering unit. There were between 36 (Denmark) and 531 (Portugal) schools per country, with an average of between 5.92 (Portugal) and 119.62 (Cyprus) students observed within each school. Across all included countries the Cronbach׳s alpha value for the CES-D was 0.82, with an average inter-item correlation of 0.43. The Cronbach׳s alpha value for self esteem was 0.82, with an average inter-item correlation of 0.31.

The country level prevalence and means of outcomes are shown in Table 2, which also indicates where countries did not provide data on an outcome. On average across all countries 60% of students had ever tried alcohol, 19% had on at least 6 occasions in the last 30 days, and 43% had drank 5 or more drinks on a single occasion in the last 30 days, 27% had ever tried a cigarette, 17% smoked at least one cigarette per day over the last 30 days, and 9% had ever tried cannabis, ecstasy or inhalants. The depressive mood scale ranged from 0 to 18 with a mean of 5.11 (SD=3.89). Country level mean depressive mood scores ranged from 3.65 in Iceland to 6.30 in Armenia. The self-esteem scale ranged from 0 to 30 with a mean of 19.54 (SD=5.15). Country level mean self-esteem scores ranged from 17.07 in Slovakia through to 21.31 in Iceland.

**Table 2:** Proportion (prop) of substance use and means of psychosocial health outcomes in each country and overall.

	Any alcohol		Regular alcohol		Binge drinking		Any smoking		Regular smoking		Any illicit drugs		Depressive mood		Self esteem	
	prop(se)	*n*	prop(se)	*n*	prop(se)	*n*	prop(se)	*n*	prop(se)	*n*	prop(se)	*n*	mean(se)	*n*	mean(se)	*n*
Armenia	0.35(0.01)	3951	0.06(0.00)	3951			0.07(0.00)	4046	0.04(0.00)	4046	0.02(0.00)	4012	6.31(0.06)	3956	21.25(0.06)	3928
Austria	0.80(0.01)	2535	0.45(0.01)	2535			0.45(0.01)	2565	0.31(0.01)	2565	0.08(0.01)	2547				
Britain	0.70(0.01)	2140	0.26(0.01)	2140	0.54(0.01)	2156	0.22(0.01)	2174	0.13(0.01)	2174	0.12(0.01)	2148	5.77(0.08)	2084	18.87(0.11)	2087
Bulgaria	0.66(0.01)	2297	0.25(0.01)	2297	0.47(0.01)	2339	0.40(0.01)	2345	0.31(0.01)	2345	0.08(0.01)	2321	5.67(0.09)	2279	19.05(0.09)	2271
Croatia	0.64(0.01)	2979	0.22(0.01)	2979	0.50(0.01)	2996	0.38(0.01)	3004	0.27(0.01)	3004	0.08(0.01)	2959	5.43(0.07)	2977	19.66(0.09)	2972
Cyprus	0.62(0.01)	6108	0.21(0.01)	6108	0.34(0.01)	6284	0.23(0.01)	6302	0.16(0.00)	6302	0.11(0.00)	6264	5.09(0.05)	6239	18.53(0.06)	6265
Czech Rep	0.76(0.01)	3844	0.24(0.01)	3844	0.52(0.01)	3889	0.41(0.01)	3898	0.25(0.01)	3898	0.19(0.01)	3828				
Denmark	0.80(0.01)	857	0.28(0.02)	857	0.60(0.02)	870	0.32(0.02)	871	0.22(0.01)	871	0.11(0.01)	855				
Estonia	0.60(0.01)	2333	0.13(0.01)	2333	0.54(0.01)	2347	0.29(0.01)	2356	0.18(0.01)	2356	0.07(0.01)	2335				
Finland	0.48(0.01)	4933	0.06(0.00)	4933	0.34(0.01)	4959	0.30(0.01)	4974	0.19(0.01)	4974	0.03(0.00)	4957	4.43(0.05)	4961		
Greece	0.71(0.01)	3044	0.23(0.01)	3044	0.41(0.01)	3044	0.22(0.01)	3057	0.14(0.01)	3057	0.05(0.00)	3046	5.43(0.07)	3044	20.83(0.11)	3041
Iceland	0.31(0.01)	3487	0.04(0.00)	3487	0.22(0.01)	3492	0.16(0.01)	3496	0.11(0.01)	3496	0.03(0.00)	3485	3.65(0.06)	3384	21.31(0.11)	3402
Ireland	0.56(0.01)	2166	0.19(0.01)	2166			0.23(0.01)	2216	0.14(0.01)	2216	0.11(0.01)	2163	5.03(0.08)	2116		
Lithuania	0.65(0.01)	2366	0.16(0.01)	2366	0.41(0.01)	2391	0.34(0.01)	2400	0.21(0.01)	2400	0.05(0.00)	2381				
Malta	0.73(0.01)	3631	0.34(0.01)	3631	0.57(0.01)	3657	0.26(0.01)	3658	0.12(0.01)	3658	0.09(0.00)	3652				
Poland	0.57(0.01)	2100	0.16(0.01)	2100	0.39(0.01)	2103	0.21(0.01)	2109	0.12(0.01)	2109	0.07(0.01)	2108				
Portugal	0.60(0.01)	3033	0.22(0.01)	3033			0.19(0.01)	3137	0.09(0.01)	3137	0.07(0.00)	3107				
Slovakia	0.63(0.01)	2428	0.20(0.01)	2428	0.50(0.01)	2457	0.37(0.01)	2467	0.24(0.01)	2467	0.14(0.01)	2420	5.48(0.07)	2417	17.07(0.08)	2422
Slovenia	0.65(0.01)	3035	0.19(0.01)	3035	0.51(0.01)	3075	0.29(0.01)	3082	0.21(0.01)	3082	0.12(0.01)	3071	4.65(0.06)	3063	18.81(0.08)	3058
Sweden	0.44(0.01)	3087	0.06(0.00)	3087	0.37(0.01)	3148	0.21(0.01)	3166	0.10(0.01)	3166	0.04(0.00)	3157				
Switzerland	0.67(0.01)	2467	0.19(0.01)	2467	0.35(0.01)	2479	0.29(0.01)	2475	0.16(0.01)	2475	0.16(0.01)	2454				
																
																
Total	0.60(0.00)	62821	0.19(0.00)	62821	0.43(0.00)	51686	0.27(0.00)	63798	0.17(0.00)	63798	0.09(0.00)	63270	5.11(0.02)	36520	19.54(0.03)	29446

Across the whole sample for all outcomes, likelihood ratio tests and intra-class correlation coefficients from the three level models (Country/School/Student) indicated that there was a significant amount of within-country between-school level variance. The estimates for the country ICCs, i.e. the similarity of students within the same country, were 0.09(0.05–0.15) for any alcohol consumption, 0.14(0.08–0.23) for regular alcohol consumption, 0.05(0.03–0.09) for binge drinking, 0.07(0.04–0.13) for having smoked, 0.09(0.05–0.16) for regular smoking, 0.10(0.05–0.17) for illicit drug use, 0.03(0.01–0.07) for depressive mood and 0.08(0.03–0.18) for self-esteem. Within the three level model, estimates for the school ICCs within countries, that is the similarity of students within the same school in the same country were: 0.14(0.10–0.19) for any alcohol, 0.20(0.14–0.28) for regular alcohol drinking, 0.11(0.08–0.15) for binge drinking, 0.15(0.11–0.19) for ever having smoked, 0.19(0.15–0.25) for regular smoking, 0.18(0.13–0.23) for illicit drug use, 0.07(0.05–0.10) for depressive mood, and 0.11(0.06–0.18) for self-esteem. For this inclusive model, the confidence intervals allow us to compare the estimates and determine whether they are statistically significantly different. The confidence intervals indicate that the ICC for depressive mood was lower in magnitude than for all substance use outcomes with the exception of binge drinking. Next, we considered country specific estimates for school ICCs.

The country specific unadjusted school level ICCs are presented in Table 3. There is considerable variability between countries in the estimates of ICCs. On the whole, ICCs were lower for psychosocial health than substance use. Of the substance use outcomes, regular smoking had the highest estimated ICC, except in 6 countries (Croatia, Estonia, Finland, Lithuania, Slovenia, and Sweden) where illicit drug use had the highest estimated ICCs. There was large variability within countries across the health outcomes. For example in Armenia ICC values ranged from 0.03(0.02–0.05) for depressive mood through to 0.18(0.10–0.30) for regular smoking.

**Table 3:** Unadjusted school ICCs.

	Any alcohol	Regular alcohol	Binge drinking	Any smoking	Regular smoking	Any illicit drugs	Depressive mood	Self-esteem
	ICC (95% C.I.)	ICC (95% C.I.)	ICC (95% C.I.)	ICC (95% C.I.)	ICC (95% C.I.)	ICC (95% C.I.)	ICC (95% C.I.)	ICC (95% C.I.)
Armenia	0.08 (0.06–0.12)	0.10 (0.05–0.19)		0.12 (0.07–0.20)	0.18 (0.10–0.30)	0.14 (0.04–0.36)	0.03 (0.02–0.05)	0.04 (0.02–0.06)
Austria	0.13 (0.08–0.20)	0.07 (0.04–0.11)		0.07 (0.04–0.11)	0.13 (0.09–0.18)	0.07 (0.03–0.17)		
Britain	0.11 (0.07–0.17)	0.07 (0.04–0.11)	0.08 (0.05–0.13)	0.05 (0.02–0.11)	0.14 (0.08–0.23)	0.06 (0.02–0.15)	0.03 (0.01–0.05)	0.01 (0.00–0.05)
Bulgaria	0.13 (0.09–0.19)	0.07 (0.04–0.13)	0.09 (0.06–0.13)	0.14 (0.09–0.19)	0.14 (0.09–0.20)	0.14 (0.07–0.24)	0.06 (0.04–0.09)	0.02 (0.01–0.05)
Croatia	0.04 (0.02–0.07)	0.07 (0.04–0.12)	0.08 (0.05–0.12)	0.07 (0.05–0.11)	0.10 (0.06–0.15)	0.14 (0.08–0.23)	0.07 (0.04–0.10)	0.02 (0.01–0.05)
Cyprus	0.00 (0.00–0.02)	0.04 (0.02–0.06)	0.03 (0.02–0.06)	0.05 (0.03–0.09)	0.08 (0.05–0.12)	0.03 (0.01–0.06)	0.02 (0.01–0.03)	0.01 (0.00–0.02)
Czech Rep	0.07 (0.04–0.11)	0.10 (0.07–0.14)	0.08 (0.05–0.11)	0.09 (0.06–0.13)	0.15 (0.11–0.20)	0.09 (0.06–0.14)		
Denmark	0.08 (0.03–0.21)	0.09 (0.04–0.19)	0.08 (0.03–0.18)	0.17 (0.09–0.29)	0.21 (0.11–0.35)	0.11 (0.04–0.27)		
Estonia	0.01 (0.00–0.09)	0.02 (0.00–0.12)	0.02 (0.01–0.06)	0.03 (0.02–0.08)	0.06 (0.03–0.12)	0.09 (0.04–0.20)		
Finland	0.04 (0.03–0.07)	0.10 (0.05–0.18)	0.08 (0.05–0.11)	0.07 (0.05–0.10)	0.07 (0.05–0.11)	0.13 (0.06–0.25)	0.04 (0.03–0.06)	
Greece	0.06 (0.03–0.10)	0.07 (0.04–0.11)	0.08 (0.05–0.12)	0.13 (0.09–0.19)	0.13 (0.08–0.20)	0.13 (0.06–0.25)	0.01 (0.00–0.04)	0.04 (0.02–0.06)
Iceland	0.04 (0.02–0.08)	0.00 (0.00–1.00)	0.03 (0.01–0.07)	0.04 (0.02–0.08)	0.07 (0.04–0.14)	0.06 (0.02–0.22)	0.05 (0.03–0.08)	0.08 (0.06–0.12)
Ireland	0.04 (0.02–0.08)	0.07 (0.04–0.14)		0.07 (0.04–0.12)	0.10 (0.05–0.17)	0.06 (0.03–0.14)	0.06 (0.04–0.10)	
Lithuania	0.04 (0.02–0.08)	0.09 (0.05–0.15)	0.04 (0.02–0.07)	0.08 (0.05–0.13)	0.16 (0.11–0.24)	0.17 (0.09–0.30)		
Malta	0.04 (0.02–0.07)	0.06 (0.04–0.11)	0.11 (0.07–0.17)	0.05 (0.03–0.10)	0.06 (0.03–0.11)	0.03 (0.01–0.09)		
Poland	0.09 (0.05–0.14)	0.04 (0.01–0.14)	0.05 (0.03–0.10)	0.09 (0.05–0.16)	0.08 (0.03–0.18)	0.07 (0.02–0.24)		
Portugal	0.07 (0.05–0.12)	0.04 (0.02–0.09)		0.16 (0.11–0.22)	0.21 (0.13–0.31)	0.12 (0.05–0.23)		
Slovakia	0.02 (0.01–0.06)	0.04 (0.02–0.10)	0.06 (0.04–0.10)	0.07 (0.04–0.12)	0.10 (0.06–0.15)	0.06 (0.03–0.13)	0.03 (0.02–0.06)	0.03 (0.01–0.06)
Slovenia	0.05 (0.03–0.09)	0.07 (0.04–0.12)	0.09 (0.06–0.13)	0.08 (0.05–0.13)	0.13 (0.09–0.20)	0.15 (0.10–0.23)	0.07 (0.05–0.11)	0.02 (0.01–0.04)
Sweden	0.07 (0.05–0.10)	0.03 (0.00–0.25)	0.05 (0.03–0.08)	0.06 (0.04–0.10)	0.09 (0.05–0.16)	0.19 (0.10–0.32)		
Switzerland	0.05 (0.03–0.10)	0.06 (0.03–0.12)	0.05 (0.03–0.10)	0.08 (0.05–0.13)	0.15 (0.10–0.23)	0.04 (0.02–0.12)		

The country specific school level ICCs adjusted for student sex and socioeconomic status are presented in Table 4. Many of the estimated ICCs were unchanged after adjustment, and regular smoking and illicit drug use still tended to have the highest ICCs and the psychosocial health variables had the lowest ICC values.

**Table 4:** Adjusted school ICC׳s.

	Any alcohol	Regular alcohol	Binge drinking	Any smoking	Regular smoking	Any illicit drugs	Depressive mood	Self-esteem
	ICC (95% C.I.)	ICC (95% C.I.)	ICC (95% C.I.)	ICC (95% C.I.)	ICC (95% C.I.)	ICC (95% C.I.)	ICC (95% C.I.)	ICC (95% C.I.)
Armenia	0.09 (0.06–0.12)	0.12 (0.06–0.22)		0.18 (0.12–0.27)	0.26 (0.16–0.40)	0.17 (0.06–0.39)	0.03 (0.02–0.05)	0.04 (0.03–0.06)
Austria	0.13 (0.09–0.20)	0.07 (0.04–0.12)		0.07 (0.04–0.12)	0.14 (0.09–0.19)	0.06 (0.02–0.16)		
Britain	0.11 (0.07–0.17)	0.06 (0.04–0.11)	0.09 (0.06–0.14)	0.06 (0.03–0.12)	0.15 (0.09–0.25)	0.05 (0.02–0.14)	0.02 (0.01–0.05)	0.01 (0.00–0.05)
Bulgaria	0.09 (0.06–0.15)	0.05 (0.03–0.11)	0.09 (0.05–0.13)	0.14 (0.10–0.20)	0.14 (0.10–0.21)	0.11 (0.05–0.22)	0.04 (0.02–0.07)	0.01 (0.00–0.05)
Croatia	0.03 (0.02–0.07)	0.06 (0.03–0.10)	0.06 (0.04–0.10)	0.07 (0.04–0.11)	0.09 (0.06–0.14)	0.15 (0.09–0.24)	0.02 (0.01–0.04)	0.02 (0.01–0.04)
Cyprus^1^	0.00 (0.00–0.02)	0.01 (0.01–0.03)	0.01 (0.01–0.03)	0.04 (0.02–0.07)	0.06 (0.03–0.09)	0.02 (0.01–0.05)	0.01 (0.00–0.02)	0.01 (0.01–0.03)
Czech Rep	0.07 (0.04–0.11)	0.10 (0.07–0.14)	0.07 (0.04–0.10)	0.07 (0.05–0.11)	0.13 (0.09–0.18)	0.08 (0.05–0.13)		
Denmark	0.08 (0.03–0.21)	0.10 (0.04–0.21)	0.08 (0.03–0.19)	0.18 (0.10–0.31)	0.21 (0.11–0.35)	0.12 (0.05–0.29)		
Estonia	0.01 (0.00–0.08)	0.02 (0.00–0.12)	0.02 (0.01–0.05)	0.03 (0.01–0.07)	0.05 (0.02–0.10)	0.09 (0.04–0.20)		
Finland	0.05 (0.03–0.07)	0.10 (0.05–0.18)	0.07 (0.05–0.10)	0.07 (0.04–0.09)	0.07 (0.04–0.11)	0.13 (0.06–0.26)	0.04 (0.02–0.05)	
Greece	0.06 (0.04–0.10)	0.07 (0.04–0.12)	0.06 (0.04–0.10)	0.13 (0.09–0.20)	0.13 (0.08–0.20)	0.10 (0.04–0.23)	0.01 (0.00–0.04)	0.04 (0.02–0.07)
Iceland	0.03 (0.01–0.06)	0.01 (0.00–0.97)	0.02 (0.01–0.06)	0.03 (0.01–0.08)	0.07 (0.04–0.14)	0.08 (0.02–0.23)	0.00 (0.00–0.00)	0.00 (0.00–0.04)
Ireland	0.04 (0.02–0.08)	0.07 (0.04–0.13)		0.05 (0.02–0.10)	0.03 (0.01–0.12)	0.06 (0.02–0.14)	0.02 (0.00–0.05)	
Lithuania	0.04 (0.02–0.08)	0.09 (0.05–0.16)	0.03 (0.01–0.07)	0.07 (0.04–0.13)	0.16 (0.10–0.24)	0.18 (0.10–0.31)		
Malta	0.03 (0.01–0.06)	0.05 (0.03–0.10)	0.10 (0.06–0.15)	0.05 (0.03–0.09)	0.05 (0.02–0.09)	0.02 (0.01–0.09)		
Poland	0.09 (0.05–0.14)	0.04 (0.01–0.14)	0.06 (0.03–0.11)	0.09 (0.05–0.16)	0.08 (0.03–0.18)	0.08 (0.02–0.25)		
Portugal	0.07 (0.04–0.12)	0.04 (0.02–0.10)		0.16 (0.12–0.23)	0.22 (0.14–0.33)	0.11 (0.05–0.23)		
Slovakia	0.02 (0.01–0.06)	0.05 (0.02–0.11)	0.05 (0.03–0.09)	0.07 (0.04–0.11)	0.09 (0.06–0.15)	0.06 (0.03–0.13)	0.01 (0.00–0.05)	0.03 (0.01–0.06)
Slovenia	0.05 (0.03–0.09)	0.05 (0.03–0.10)	0.09 (0.06–0.14)	0.09 (0.05–0.13)	0.14 (0.10–0.21)	0.16 (0.10–0.24)	0.02 (0.01–0.04)	0.02 (0.01–0.04)
Sweden	0.07 (0.05–0.11)	0.03 (0.00–0.23)	0.05 (0.03–0.08)	0.06 (0.04–0.11)	0.08 (0.04–0.16)	0.19 (0.10–0.33)		
Switzerland	0.05 (0.02–0.10)	0.06 (0.03–0.12)	0.05 (0.03–0.10)	0.08 (0.05–0.13)	0.15 (0.09–0.22)	0.04 (0.01–0.12)		

1 Parental education not available in this country.

## 4 Discussion

We aimed to provide estimates of school level ICCs for substance use and psychosocial health outcomes in twenty one European countries. We compared the estimates within countries, and across countries to determine the extent of the differences within countries by outcome, and between countries within the same outcome.

We found that ICCs for health outcomes in 15 year olds in schools varied substantially within European countries across health outcomes, and between countries for the same health outcomes. There were higher ICCs for substance use, particularly regular smoking and any illicit drug use in the last 30 days, than for measures of psychosocial health. The within country ICCs for health outcomes varied by changes in the measurement of particular health outcomes, for example the ICCs for regular smoking were higher than the ICCs for having smoked at all in the last month. This suggests that researchers using ICCs for power calculations in CRCTs, or for adjustments in meta-analyses, should use ICCs specific to the outcomes they intend to measure in the way they intend to measure them.

Our findings of higher ICCs for substance use compared to psychosocial health outcomes is consistent with the current literature (Hale et al., 2014, Sellström and Bremberg, 2006). The comparatively high school-level variation for substance use and low school-level variation for psychosocial health may be explained by the relative importance of peer influences on such behaviours. Moffitt (1993) posits that substance use arise partly as a result of social mimicry, in which adolescents re-enact the risk-taking behaviours of their peers to gain social acceptance. The visibility of a small number of students within a given school displaying substance use could lead to a proliferation of such behaviour within that school. Psychosocial health outcomes such as depressive mood are less overtly visible to peers than substance use, and may therefore be less susceptible to social mimicry.

A related concept is that of “peer contagion” effects, whereby problem behaviours are amplified by affiliations with similar peers (Cohen and Prinstein, 2006, Dishion and Tipsord, 2011). Changes in substance use and emotional wellbeing are the unintended consequences of relationships with peers. The influence of peers on health substance use is well-established, but an emerging literature indicates that peer influence is important for internalizing problems such as depressive mood also (Brechwald & Prinstein, 2011). Although the relative importance of peer effects for different outcomes has not been established.

An alternative explanation for the higher ICCs for risk taking behaviours focuses on the schools rather than the students. Markham and Aveyard׳s (2003) theory of health promoting schools indicates that these risk taking behaviours represent a rejection of the schools values and consequent affiliation with youth subculture. Many of our findings are consistent with this theory. The theory suggests ICCs should be higher for behaviours that are most ‘deviant’ and suggestive of anti-school rebellion such as drug use and regular smoking, with smaller ICCs for more normative behaviours such as experimentation with alcohol and tobacco. However, our finding that regular drinking does not consistently have higher ICCs than ever having tried alcohol is inconsistent with this theory.

A key policy question emerging from the higher estimated ICCs for substance use compared to psychosocial health outcomes is whether CRCTs in school settings are likely to be more effective for risk taking behaviours than for psychosocial health given the higher proportion of variance at the school level. Our findings suggest the answer could be yes, although there is insufficient evidence to form any firm conclusions. It could also be the case that the higher ICCs for substance use are due selection effects into schools and subsequent compositional differences rather than contextual effects (Macintyre et al., 2002). However, given the ICCs are different for different health outcomes, and that the same patterns pertain for unadjusted and adjusted ICCs, it is unlikely that the high ICCs for substance use is merely due to compositional differences.

There is not enough available evidence in the wider literature to determine if health risk behaviours are more amenable than psychosocial health to school level interventions. A high quality systematic review by Langford et al. (2014) of health promoting school interventions (school-level interventions that focussed on altering the school environment/ethos in addition to changing the curriculum and building links with families or communities) found significant effects of interventions for smoking, but less conclusive evidence of effects for drinking and drug use (because of an insufficient number of studies) (Langford et al., 2014). The two included mental health interventions did not have significant benefits. Whilst this is consistent with our findings, there is clearly not enough evidence to determine for which outcomes school based interventions are most effective.

Furthermore, evidence from multilevel models may be influenced by the paradoxical situation whereby a lower proportion of the variance at the school level results in a higher likelihood of obtaining a “significant” association between a school level variable (such as an intervention) and the outcome (Lagerlund et al., 2015, Merlo, 2015). That is, the less that the school context matters for explaining variance in the outcome, the easier it is to find a “significant” association between the two. The ICC values presented here suggest that, in most cases, caution needs to be taken when interpreting the association between school-level variables and the psychosocial health outcomes.

Nevertheless, the mounting evidence from observational and intervention studies that schools influence student health has resulted in extensive research and policy interest in using school-based interventions to improve young people׳s health (Department of Health, 2009, Hale and Viner, 2012, National Institute for Health and Clinical Excellence, 2010). This reflects a greater understanding of the potential for place to effect people, and of the potential utility of CRCTs (Klar and Donner, 2001, Raudenbush, 1997). Knowledge of the causal mechanisms driving the ICC estimates is not necessary for the ICCs to be useful for informing sample size estimations in CRCTs, or for making adjustments to regression estimates.

There are several strengths to this paper; we considered substance use and aspects of psychosocial health, we included a large number of countries, the samples were nationally representative and the sample sizes of schools within countries and students within countries were mostly large. However, the number of students selected per school is variable, and where the average number of students per school is low (for example Portugal (5.62)), the ICC value is less reliable. We were limited in the scope of this paper because there was no available information on schools so we were unable to consider whether the ICCs are explained by contextual or compositional effects. We did not consider changes in ICC values along differential values of student level variables (Merlo, Yang, Chaix, Lynch, & Råstam, 2005). For example it may be the case that ICC׳s for substance use are higher amongst students in low socioeconomic group and lower amongst high socioeconomic groups. We also did not have information on important student level demographic characteristics related to the outcomes such as ethnicity or religion. Furthermore, all participating students were between the ages of 15–16 and so we are unable to consider changes in the ICC׳s with age. A final limitation was that we did not consider the class level, however this may not be important as students of this age group spend little time in their form classes and class compositions change for each subject.

## 5 Conclusion

CRCTs are increasingly utilised to assess the impact of interventions, especially in school settings. For school level ICCs to be effectively utilised in informing sample size requirements for CRCTs and adjusting estimates from meta-analyses, the school level ICCs need to be both country and outcome specific. Whether some health outcomes are more malleable to school-level intervention, and whether this differs across countries, are key policy issues requiring further research.

## Funding

This research was supported in part by a small research grant from the British Academy (SG142822). The Policy Research Unit is funded by the Department of Health Policy Research Programme, England. This is an independent report commissioned and funded by the Department of Health. The views expressed are not necessarily those of the Department.

## Conflict of interest

None declared.
